# Intervention among Suicidal Men: Future Directions for Telephone Crisis Support Research

**DOI:** 10.3389/fpubh.2018.00001

**Published:** 2018-01-19

**Authors:** Tara Hunt, Coralie J. Wilson, Alan Woodward, Peter Caputi, Ian Wilson

**Affiliations:** ^1^School of Medicine, University of Wollongong, Wollongong, NSW, Australia; ^2^Illawarra Health and Medical Research Institute, Wollongong, NSW, Australia; ^3^Centre for Mental Illness in Nowra District: Goals and Prevention (MINDtheGaP), Nowra, NSW, Australia; ^4^Centre for Mental Health, University of Melbourne, Melbourne, VIC, Australia; ^5^Lifeline Research Foundation, Lifeline Australia, Canberra, ACT, Australia; ^6^Suicide Prevention Australia, Sydney, NSW, Australia; ^7^School of Psychology, University of Wollongong, Wollongong, NSW, Australia

**Keywords:** men, suicide, suicide intervention, crisis intervention, telephone crisis line, telephone helpline

## Abstract

Telephone crisis support is a confidential, accessible, and immediate service that is uniquely set up to reduce male suicide deaths through crisis intervention. However, research focusing on telephone crisis support with suicidal men is currently limited. To highlight the need to address service delivery for men experiencing suicidal crisis, this perspective article identifies key challenges facing current telephone crisis support research and proposes that understanding of the role of telephone crisis helplines in supporting suicidal men may be strengthened by careful examination of the context of telephone crisis support, together with the impact this has on help-provision for male suicidal callers. In particular, the impact of the time- and information-poor context of telephone crisis support on crisis-line staff’s identification of, and response to, male callers with thoughts of suicide is examined. Future directions for research in the provision of telephone crisis support for suicidal men are discussed.

## Introduction

International mental-health policy has highlighted the role of telephone crisis support in comprehensive suicide intervention systems as a necessary provider of immediate, anonymous, accessible, and cost-effective crisis intervention (1–4). This emphasis highlights the importance of establishing the efficacy and service outcomes of telephone crisis support in vulnerable communities. To date, little research has focused on telephone crisis support among men—a population group that has a higher prevalence of mortality from suicide than other groups (5, 6). Highlighting areas for future research, this perspective article identifies key challenges facing current telephone crisis support research and proposes that understanding the role of telephone crisis helplines in supporting suicidal men may be strengthened by careful examination of the context of telephone crisis support, together with the impact this has on help-provision. To that end, this perspective specifically focuses on the potential impact that the time- and information-limited telephone crisis support context has on the provision of care to men who are currently suicidal.

## Suicide Risk Among Men

Suicide is a leading cause of death around the world (2). Global estimates report that in 2015, 10.7 people per 100,000 took their own life by suicide (7). These statistics are likely to underreport the true incidence, with cultural taboos, criminalization of attempted suicide, and record keeping procedures impacting the statistical recording and reporting of suicide (8). From the records available, it is evident that there are consistent sociodemographic patterns associated with the prevalence of suicide deaths. In high-income western countries, such as Australia, USA, New Zealand, and Canada, suicide prevalence tends to be higher than low- and middle-income countries (12.7 compared to 11.2 per 100,000 population) (2). In high-income countries, men account for around three times as many deaths by suicide as women, but the gender ratio reduces to 1.5 men for each woman in low- and middle-income countries. The consistency of the gender-ratio in suicide prevalence across higher-income countries, suggests there may be common social and cultural factors that contribute to the suicide ratio across countries. These factors may include availability of different means of suicide, gender differences in the acceptability of self-destructive behavior between men and women, and constructions of masculinity and associated beliefs about the appropriateness of help-seeking for suicidality (9–11). There is also research reporting that help-seeking practices among men are varied (12, 13), with some men avoiding mental-health services, but others actively seeking help from a variety of sources, including family, friends, GPs, and allied health services who can redirect suicidal men to mental-health services (13–15). The unique characteristics of help-seeking practices among suicidal men position telephone crisis support as a viable option for identifying and responding to suicidal males in a way that facilitates further help seeking.

## Is Telephone Crisis Support Attractive for Suicidal Men?

Telephone crisis support services have been developed and implemented around the world to provide support to people experiencing crisis, which is defined as a time-limited state of psychological disequilibrium that surpasses the person’s current resources and coping ability (16–18). The experience of crisis may be related to a wide range of issues, including mental-health problems (e.g., depression), navigating social, emotional, and environmental stressors, or the experience of social isolation and loneliness. Telephone crisis lines often have a particular focus on identifying and responding to suicidal individuals, as life stressors can trigger suicidal crisis, particularly when combined with pre-existing mental-health conditions, such as depression (19–21). Crisis lines are usually staffed by paid and unpaid volunteers and para-professionals who are trained in crisis and suicide intervention (16, 22). This includes guidelines on the identification of callers in a suicidal state and strategies to reduce callers’ current experience of crisis and/or suicidal states, and to enhance the safety of callers by referring to ongoing support (23). By their nature, calls to telephone crisis helplines are often anonymous and are often intended to be a once-off intervention (24).

Statistics describing mental-health service usage suggest that men who seek help for mental-health problems access telephone crisis support at comparable rates to other sources of psychological support (25, 26). For example, from 2012 to 2013, men accounted for approximately 40% of callers who accessed the four main national Australian helplines (25) and 33% of clients who accessed psychologists and other allied health services (27). Although it is not known whether the rate of suicidal men who accessed a telephone crisis support was similar to that for the other professional mental-health services, these statistics suggest that telephone crisis support services can be attractive to a large proportion of men dealing with at least some types of mental-health problems, many of which co-occur with suicide risk.

Potential reasons for the attractiveness of telephone crisis support for males include having a confidential environment, accessibility regardless of geographical location, a lack of screening or entry assessment, the ability to make and terminate the call at any time, and person-centered, collaborative problem-solving support approaches [e.g., Ref. (28, 29)]. Providing a question for future research, it is possible that these characteristics are equally attractive to suicidal men. If so, it highlights the importance of research to identify (i) ways to optimize telephone crisis support for males, (ii) areas and strategies for ongoing gender-specific training to upskill those who provide telephone crisis support for males, and (iii) ways to improve policy relating to telephone crisis support for suicidal males.

## Evidence for Telephone Crisis Support

Telephone crisis support services are widely promoted (1), yet evidence attesting to the efficacy of telephone crisis support services is less than equivocal. This may be in part due to the anonymous nature of the service and the methodological and ethical restrictions associated with evaluating intervention effectiveness in a suicidal population (30, 31). Ethical issues preclude traditional pre-post follow-up designs in populations that are acutely suicidal, which may otherwise evaluate the efficacy of telephone crisis support. Recently, Hvidt et al. (6) conducted a systematic literature review which examined efficacy studies that were published over the past 45 years to determine whether current research supports the use of telephone crisis intervention with suicidal callers. By examining all relevant outcome measures, Hvidt et al. (6) provided a comprehensive overview of the impact of telephone crisis support services on suicidal callers. The review found 18 articles meeting inclusion criteria that suggested telephone crisis lines can be effective in reducing imminent suicide risk for both male and female callers, at the time of the call [e.g., Ref. (17, 23, 31, 32)]. This finding was also balanced with the caution that the strength of current research is limited due to “sub-optimal designs and outcome measures” (p. 156), which needs to be rectified in future telephone crisis support research.

Another question for future research is to carefully examine whether the context of telephone crisis support impacts the help that suicidal callers can be provided. In the development of suicide prevention technologies, methods of intervention are often transposed from one modality to another, such as from the application of face-to-face support methods to the telephone crisis support context, or from telephone crisis support methods to Internet chat-based services (33). The validity and reliability of research results might be hampered if the research design does not assess how these different contexts impact the effectiveness of commonly used telephone crisis support procedures. For example, research needs to examine the impact of the telephone context on the identification of suicide risk among male callers who are unable or unwilling to express suicidality. Investigating the impact of context on telephone crisis support procedures may be facilitated by collaboration between researchers and telephone crisis services using a codesign process to produce research that reflects both scientific rigor and the needs of the organization. Codesigned research has been shown to produce results that are directly and efficiently translatable into service outcomes (34–36).^1^ Understanding how telephone crisis support is distinct from other mental-health support modalities is likely to facilitate a clearer understanding of the conditions under which telephone crisis support is appropriate and effective, particularly for men.

## Factors Impacting the Support of Suicidal Men in the Telephone Crisis Support Context

The capacity of telephone crisis workers (TCWs) to identify and assist suicidal male callers quickly and effectively is likely to be challenged by the time-limited telephone crisis line context. Traditionally, suicide assessment and intervention in face-to-face counseling and other sources of professional help, depend on verbal and non-verbal communication skills. Non-verbal communication contributes to building both the relationship and mutual understanding between counselor and male clients, which is vital in successful suicide intervention (37–39). Valuable information suggesting the possibility of current suicidality can be relayed in non-verbal cues (40, 41). Non-verbal communication also serves a wide range of functions in the clinical setting. This is particularly through the regulation of the therapeutic interaction by establishing rules such as turn-taking and showing sensitivity to changes in the relationship (42), conveying vital information, such as empathy (37) and understanding (43), and enhancing rapport—particularly with male clients—through portraying interest and engagement with the client’s intentions, issues, and emotions (39), reassuring the client that the counselor is trustworthy.

In telephone crisis support, the non-verbal communication that may support the accurate identification of males callers in suicidal crisis is limited, which means TCWs largely rely on verbal expressions of suicidal intent (44, 45). This reliance may compromise the accurate identification of suicidality if callers are unable or unwilling to describe their suicidal state. A retrospective case-file audit of 157 suicidal patients found that up to two-thirds of patients denied having suicidal ideation when last asked, yet half died by suicide within 2 days (46). Advances in neuroscience research provide some evidence to suggest that suicidal ideation and behavior indicate the presence of brain changes that physically impair the suicidal individual’s cognitive ability to articulate how they are feeling (47, 48). This impairment can lead to a lack of disclosure (49, 50), and is a common problem among suicidal men who have reported impairment in the ability to articulate feelings of distress, despair, and suicidal ideation (51, 52).

If a caller is unable or unwilling to verbally express suicidal intent, TCWs are dependent on other components of verbal communication to identify whether a caller is suicidal before making support-relevant decisions. Components may include silence, inflection, tone, timbre, intensity, and speed (44). In the absence of direct statements of suicidal intent, TCWs may also rely on information such as callers’ background or characteristics (e.g., callers’ gender) to make inferences about the likelihood that callers are suicidal and to guide interactions with the caller (53, 54). For example, whether a caller is male or female may shape the information that TCWs listen for to identify the presence of suicidal intent. This type of information processing short-cut may be highly efficient (55, 56), but only in so much as the TCWs’ knowledge and expectations of signs of suicidal presentation in male and female callers are accurate. The telephone context requires TCWs to be specifically trained in strategies and techniques to identify suicide risk from non-verbal cues and signs that each suicidal caller provides.

## Recognizing Correlates of Suicide Risk Among Men

While telephone supporters are often purposefully trained *not* to conduct clinical assessment or diagnosis of specific psychiatric conditions that occur with suicidal ideation and behavior (e.g., Lifeline Australia, Suicide Prevention Lifeline USA), it is still important for TCWs to be able to recognize and respond to correlates of suicide, including those that may indicate the presence of psychiatric conditions. For example, cannabis use or dependence can be critically involved in promoting suicide risk with co-occurring psychiatric conditions (57), and acute alcohol use and negative life-events predict proximal increases in suicidal ideation immediately preceding suicide attempts (58). Results of a recent systematic review of the relationship between alcohol consumption and suicidal behavior suggest that the association between alcohol consumption and suicidal behavior is stronger among men compared to women, although this is subject to cultural variation (59). Given that emergent research suggests that the suicidal process has time-limited periods of acute intensity (60–62), near-term predictors of suicide attempts, such as substance use, which are often detected through telephone crisis support, may make it easier to detect current suicide risk among callers—particularly suicidal male callers. Due to the difficulties involved in relying on verbal statements of suicidal intent, having the potential for increased access to callers’ information about existing correlates of suicide risk highlights the need for TCWs to also be trained in ways to identify non-verbal signs of psychiatric conditions that commonly co-occur with suicide risk.

## Is Gender Bias Promoted by the Telephone Context?

In the absence of the callers specifically stating their suicidal intent, TCWs identification and interpretation of callers at risk for suicide from suicide signs is likely to involve pattern recognition (63). By definition, pattern recognition is not a conscious or critical thinking process (64). Instead, it occurs when an individual sub-consciously recognizes (accurately or erroneously) relationships between features of a problem, which in turn, cue memories about that problem and how to manage the problem (65, 66). The use of accurate pattern recognition is adaptive within time- and information-limited contexts (67, 68) and is often highly effective (56, 69), which may also be the case for TCWs. However, basing decision-making on erroneous pattern recognition may lead to selective attention of features in a call that mislead the TCW’s subsequent interpretation of, and response to, the caller’s actual presentation (55). This may take the form of asking questions about suicide that are not best matched to the individual needs of the caller. For example, research has found that TCWs identify different patterns of suicide signs that are associated with suicide risk in male and female callers, which may contribute to under-identification of suicidal callers if they present differently to the TCWs’ expectations.^2^ Alternatively, if a TCW predicts that a caller is suicidal but unlikely to talk about their intent, the TCW might appeal to authority and question, “We have to ask this to everybody, but do you have thoughts of harming yourself?” which may inadvertently minimize the callers’ experience of crisis support (70).

There are implications for the provision of care to suicidal male callers if TCWs use misinformed or biased pattern recognition to support decision-making. In particular, research has found different associations between gender and suicidal behavior in men and women that may influence TCWs’ interpretation of callers’ suicidal presentation (71–73). Focus group interviews with young people found that they associated suicide in men with strength, courage, honor, impulsivity, and decisiveness; whereas, young people associated suicide in women with love and relationships, manipulation and revenge, and a plea for help (73). These associations appear to be broadly shared and consistent with patterns identified in the narratives of the deaths of men and women that are reported by friends and relatives (74), as well as in media reporting about suicide (75, 76). It is possible that gender-based associations of male and female suicidal behavior influence TCWs’ expectations about suicidal presentation, impacting sub-conscious pattern-recognition processes. If accurate pattern recognition fails due to TCWs’ expectations about male callers’ suicidal presentation, men at risk of suicide may not be identified and vital intervention opportunities may be missed, with potentially fatal consequences. The same may be also true for suicidal female callers.

## Future Directions for Telephone Crisis Support Research

The challenges that are described in this perspective, including the restricted time and information context, highlight several directions for future research that have potential to enhance service delivery. First, the telephone crisis support context may restrict TCWs’ capacity to identify suicidal callers if they are unable or unwilling to express suicidal intent. In the absence of verbal statements of suicidal ideation, TCWs may rely on pattern recognition of suicide signs to identify imminent suicidal risk (63, 77). The reliability of currently circulated signs of suicide has been questioned (78, 79) and points to the need for future research to determine whether signs of suicide that are currently used in suicide intervention training are sufficient to effectively and accurately identify imminent suicidality in callers. Further, the impact of gender and gender practices on the expression and interpretation of mental-health issues (80) highlight the need to determine the extent to which gender impacts TCWs’ interpretation of, and response to, signs of suicide and co-occurring disorders that proximate suicide risk in callers. Understanding how signs of suicidal distress are identified, interpreted, and responded to by TCWs is of vital importance for ensuring that telephone crisis-line services are able to respond effectively and appropriately to suicidal crisis. In particular, future research needs to examine whether TCWs’ compensate for the unique difficulties that are associated with the telephone crisis support context through decision-making shortcuts, such as pattern recognition, and how this impacts TCWs’ interpretation of, and subsequent response to, male callers’ suicidal presentation. By addressing these issues, this research would contribute to training that supports TCWs’ in treating each caller as separate and unique, regardless of similarities with previous callers, or expectations of suicidal presentation between various groups, including males and females, which are established in the research literature and repeated in the mass media.

## Conclusion

Telephone crisis support offers an important opportunity to identify and support suicidal men. However, there is little research that has examined TCWs’ interpretation of, and response to, callers with suicidal presentation while considering the unique time- and information-limited context of telephone crisis support. This suggests that TCWs are reliant upon callers’ verbal statements of suicidal intent and other proximal signs to identify a caller as imminently suicidal. The unique context of telephone crisis support may particularly impact TCWs’ provision of care to suicidal men by introducing potential bias into TCWs’ interpretation of, and response to, caller presentation. The recognition of these unique barriers and facilitators in telephone crisis intervention research may be enhanced through: (i) research investigating the impact of gender on TCWs’ interpretation of, and response to, male callers with suicidal presentation that is specifically designed to address the weaknesses in existing literature, (ii) the translation of these findings into TCW education, (iii) and incorporating these findings into service policy and procedures. Importantly, enhancing the appropriateness of telephone crisis support for suicidal men may result in increased word-of-mouth referrals to the service by targeting the 40% of men who become suicidal but do not seek any form of support (81–84). It is possible that men who experienced positive outcomes from the support provided by a telephone crisis line will describe their experience or provide suggestions for recognizing the need for help to other men in their social or professional networks. Developing an understanding of how TCWs respond to men experiencing suicidal crisis is an important step toward enhancing the effectiveness of telephone crisis support for suicide intervention.

## Ethics Statement

This article does not contain any studies with human participants or animals performed by any of the authors. Informed consent: consent was not required as this article does not contain any studies with human participants or animals performed by any of the authors.

## Author Contributions

TH, CW, PC, IW, and AW contributed to the design of the work; TH drafted the first version of the manuscript and CW, PC, IW, and AW revised it critically for important intellectual content, approved for the final version to be published, and agreed to be accountable for the work.

## Conflict of Interest Statement

The authors declare that the research was conducted in the absence of any commercial or financial relationships that could be construed as a potential conflict of interest.

## Footnotes

^1^Hunt T, Wilson C, Woodward A, Caputi P, Wilson I. A community-academic partnership model for suicide prevention research. (Manuscript in preparation, 2017).

^2^Hunt T, Wilson C, Caputi P, Wilson I, Woodward A. Patterns of signs that telephone crisis support workers associate with suicide risk in telephone crisis line callers. (Manuscript submitted for publication, 2017).
